# 551 Age Does Not Predict Admission Culture Positivity

**DOI:** 10.1093/jbcr/irac012.179

**Published:** 2022-03-23

**Authors:** Anastasiya Ivanko, Kathleen S Skipton Romanowski, Angela Man, Joyce E Wall, Najib M Allabadi, Marc G Jeschke, Alisa Savetamal, John T Schulz

**Affiliations:** UC Davis Medical Center, Sacramento, California; UC Davis, Sacramento, California; Massachusetts General Hospital, Boston, Massachusetts; Bridgeport Hospital, Seymour, Connecticut; Vohra physician group, Fresno, California; U of T, Toronto, Ontario; Yale New Haven System/Bridgeport Hospital, Bridgeport, Connecticut; Massachusetts General Hospital, Boston, Massachusetts

## Abstract

**Introduction:**

Burn patients are susceptible to wound infections, urinary tract infections, pneumonia, and bloodstream infections. With rising rates of community colonization with multidrug-resistant organisms (MDRO), the colonization of wounds with commensal organisms is more concerning than ever, which is particularly true in patients with recent hospital admissions, advanced age, or institutional living situations. The purpose of this study was to examine if age was a factor in obtaining admission cultures and if older patients were more likely to have positive cultures.

**Methods:**

A retrospective chart review was conducted involving burn patients admitted at three ABA verified burn centers from January 2016 - December 2017. Data collected included demographics, burn injury, and cultures obtained within 24 hours of admission. Patients were divided into 10-year age increments from 20 to ≥ 80 years old. Data analysis was conducted using Chi-square, Fisher Exact, and Kruskal-Wallis tests.

**Results:**

A total of 1615 patients (mean age 45.9± 17.7 years, 1145 males (70.9%), mean burn size (TBSA) 9.6± 14.2%) were analyzed. Admission cultures obtained were: 656 (40.6%) wound cultures, 196 (12.1%) urine cultures, 139 (8.6%) blood cultures, and 1445 (89.5%) Methicillin-Resistant Staphylococcus Aureus (MRSA) screen. In all age groups, there were no significant differences between patients who had wound cultures (p = 0.97), blood cultures (*p* = 0.39), or MRSA screening (*p* = 0.9). As patients aged, they were more likely to have urine cultures obtained (p=0.01); - 23% of patients >80 years old had urine cultures ordered at admission compared to 8.6-16.9% of younger patients. Positive results by age group: wound cultures (p= 0.09), urine cultures (p= 0.16), blood cultures (0.10), MRSA screen (p=0.98). In looking at increased exposure to MDROs prior to admission by age groups, patients in the 61–70year (8.33%), 71–80-year (5.68%), and >80-year (6.67%) age groups were more likely to have a recent (within 30 days) hospitalization (*p* = 0.02), but there was no significant difference in pre-hospital institutionalization (i.e., prison, skilled nursing facility) by age group (*p* = 0.06). With a recent hospitalization, MRSA screening was more likely to be positive (11.3% vs. 4.9%, *p* = 0.05).

**Conclusions:**

All burn patients are susceptible to infections. Urine cultures were more likely to be obtained in older burn injured patients who are 80 years of age or older. There was no significant difference in culture positivity by age. Apart from MRSA screen positivity, there was no increased risk of urine, wound, or blood culture positivity with recent hospitalization or institutionalization. The utility of screening all patients for MDROs on admission should be considered for patients 20 years of age and older.

